# Circulating Placental Growth Factor as a Prognostic Biomarker in High-Risk Glioblastoma Patients

**DOI:** 10.3390/biomedicines14071628

**Published:** 2026-07-20

**Authors:** Filippo Gagliardi, Francesca Roncelli, Silvia Snider, Pierfrancesco De Domenico, Daniela Boselli, Simona Di Terlizzi, Chiara Villa, Pietro Mortini

**Affiliations:** 1 Department of Neurosurgery and Gamma Knife Radiosurgery, IRCCS San Raffaele Scientific Institute, 20132 Milan, Italy; 2 Vita-Salute San Raffaele University, 20132 Milan, Italy; 3 Tissue Dynamics and Biomarker Signature Discovery, San Raffaele Telethon Institute for Gene Therapy, IRCCS San Raffaele Scientific Institute, 20132 Milan, Italy; 4 FRACTAL—Flow Cytometry Resource, Advanced Cytometry Technical Applications Laboratory, Vita-Salute San Raffaele University, 20132 Milan, Italy

**Keywords:** glioblastoma, angiogenesis, blood–brain barrier, prognostic biomarker

## Abstract

**Background/Objectives:** Angiogenesis in glioblastoma (GBM) is a multifactorial process, and blood–brain barrier disruption enables the detection of circulating mediators. The clinical relevance of circulating placental growth factor (PlGF) in GBM remains unclear. This study aimed to investigate the role of PlGF in GBM and its association with disease characteristics and outcomes. **Methods:** We conducted a prospective observational study on 54 patients with IDH-wildtype GBM. Plasma samples collected at diagnosis and recurrence were analyzed using a multiplex panel of angiogenesis mediators. Associations with clinical, radiological, molecular, and treatment-related variables were assessed, along with survival outcomes. Statistical analysis was performed with R 4.5.0. **Results:** At baseline, PlGF correlated with multiple angiogenic mediators, including VEGF, IL-6, angiopoietin-1, EGF, FGF, IL-8, and TNF-α. Higher PlGF levels were associated with radiopathological features of tumor biology, including proliferation markers and the FLAIR/contrast enhancement ratio. In high-risk patients (RPA 3–4; *n* = 33), low baseline PlGF identified a subgroup with significantly longer overall survival (17.6 vs. 8.5 months; log-rank *p* = 0.031) and retained a protective association in multivariable models. In the overall cohort, this association was weaker and did not reach statistical significance. Exploratory longitudinal analyses suggested an increase in PlGF at recurrence in selected molecular and treatment-defined subgroups, while no association with bevacizumab exposure was observed. **Conclusions:** Circulating PlGF may reflect tumor biology in GBM and shows prognostic relevance in high-risk patients, where low baseline levels identify a subgroup with improved survival. These findings support PlGF as a candidate circulating biomarker and warrant validation in larger prospective cohorts.

## 1. Introduction

Angiogenesis in glioblastoma (GBM) is a multifactorial process involving cytokines, interleukins, growth and transcription factors, and membrane molecules. These mediators act both independently and interdependently through shared intracellular signaling pathways, predominantly via autocrine and paracrine mechanisms [1,2].

Blood–brain barrier (BBB) disruption, however, allows these mediators to spill over into the systemic circulation, allowing for quantification of these mediators and raising the possibility of a concomitant endocrine action [3].

Angiogenesis mainly involves mechanisms of new vessel formation, sprouting, vascular permeabilization, and evolution in angioarchitecture, and it is often associated with onco-permissive immunomodulation and tissue remodeling (epithelial-to-mesenchymal transition, EMT).

Interest in characterizing angiogenic mediators has increased to identify potential biomarkers and therapeutic targets. However, the role of Placental Growth Factor (PlGF) in GBMs remains largely unexplored.

PlGF plays a key role in placental pathology (e.g., eclampsia) [4], vitreoretinal diseases (e.g., diabetic retinopathy and macular disease) [5], and selected oncological settings (e.g., gastric cancer) [6].

This study aims to characterize the role of PlGF in GBM angiogenesis and progression by quantifying its plasma levels at diagnosis and recurrence, assessing its interplay with other angiogenic mediators, and analyzing correlations with demographic, radiological, pathological, and biohumoral variables in relation to oncological outcomes.

## 2. Material and Methods

### 2.1. Plasma Sample Collection and Analyses

Patients were prospectively enrolled between September 2019 and July 2023 based on the date of surgery. Clinical follow-up was available until February 2025, corresponding to a maximum follow-up of 66 months. Fifty-four plasma samples were collected from patients at the Neurosurgery Department, Ospedale San Raffaele (Milan, Italy), after written informed consent and in accordance with the Declaration of Helsinki. The study was approved by the Comitato Etico Territoriale LOMBARDIA 1 (Regional Ethics Committee, Lombardy, Italy) with the protocol code NCH02-2022 (13 July 2022).

We included adults (≥18 years) with histologically confirmed IDH-wildtype glioblastoma, CNS WHO grade 4, who were candidates for surgery with the intention of maximal safe resection either at diagnosis or at first recurrence, and who were planned for adjuvant therapy according to standard of care. We excluded patients eligible for biopsy only, those enrolled in interventional clinical trials at the time of sampling, and cases with non-GBM histologies.

This was a prospective observational cohort; no randomization or allocation concealment was performed. Treatments followed standard-of-care clinical practice and treating clinicians were not blinded. Staff quantifying PlGF and the angiogenesis panel worked with coded sample IDs and had no access to clinical outcomes during analysis. Statistical analyses were conducted on a de-identified dataset; survival outcomes (OS/PFS) were extracted from electronic records. No additional blinding procedures were implemented. No participants were excluded after enrollment. All 54 patients contributed to the baseline analyses; the number included in each model is reported in the corresponding tables. Survival analyses included all 54 patients.

Samples obtained for routine diagnostic/monitoring purposes were processed and stored by the institutional biobank, Biological Resource Center (CRB-OSR, Num ID CRB in BBMRI-ERIC: bbmri-eric: ID:IT 1383758011993577:collection:e5b5e707eb1a416).

The LEGENDplex™ Human Angiogenesis Panel 1 (10-plex; Biolegend, San Diego, CA, USA; Cat. No. 741215) was used for simultaneous measurement of IL-6, Angiopoietin-1, Angiopoietin-2, EGF, FGF-basic, CXCL-8 (IL-8), PECAM-1 (CD31), PlGF, VEGF, and TNF-a, according to the manufacturer’s instructions. Samples were analyzed in duplicate on a BC CytoFLEX S (Beckman Coulter, Brea, CA, USA), and data were processed with LEGENDplex™ Data Analysis Software (BioLegend, San Diego, CA, USA).

### 2.2. Retrospective Data Collection

We recorded demographics (age, sex), Karnofsky Performance Status (KPS), comorbidities, and calculated Recursive Partitioning Analysis (RPA) classes [7,8]. Current steroid and antiepileptic therapy at sampling was annotated. Radiological variables included lesion location/side, multifocality, and volumetrics computed with BRAINLAB^TM^ software. Volumes were segmented on Fluid Attenuated Inversion Recovery (FLAIR) and contrast-enhancing (CE) sequences; derived volumes included FLAIR minus CE, CE minus necrosis, and the FLAIR-to-CE ratio.

Extent of resection (EOR) was assessed on early postoperative imaging obtained within 48 h of surgery: post-contrast T1-weighted brain MRI was the preferred modality; when MRI was unavailable or contraindicated, a contrast-enhanced CT was used. EOR was classified according to RANO as gross-total resection (no residual contrast-enhancing disease), or subtotal resection (any residual contrast-enhancing tumor) [9]. Non-enhancing FLAIR hyperintensity adjacent to the cavity was not considered residual measurable disease.

Treatment-related variables comprised the extent of resection and adjuvant treatments. A binary covariate “Stupp enrollment” was derived from “Stupp start”; to minimize immortal time bias, exposure was anchored at diagnosis (intention-to-treat indicator).

### 2.3. Statistical Analyses

#### Analyses Were Performed in R 4.5.0

Normality of continuous variables was assessed with the Shapiro–Wilk test. Descriptive statistics are reported as mean ± SD (normal data) or median and IQR (non-normal data); categorical data as counts and percentages. Two-group comparisons used Student’s *t*-test or Mann–Whitney U test, as appropriate. For multi-group comparisons we used one-way ANOVA (normal data) or Kruskal–Wallis (non-normal data). Categorical variables were compared using χ^2^ or Fisher’s exact test; ordinal scores (e.g., neurological scales) were summarized as median (IQR) and analyzed with non-parametric statistics. Correlations between continuous variables were evaluated with Spearman’s rho.

Survival analyses employed univariable and multivariable Cox proportional hazards models. PlGF was dichotomized using the maximally selected rank statistic. Cox models for overall survival (OS) were fitted in the full cohort and in the RPA 3–4 subgroup. RPA stratification was included a priori as a clinically meaningful classification to explore whether the prognostic role of PlGF differed across risk categories. This approach was intended to assess whether the prognostic signal of PlGF could be better captured within a more homogeneous high-risk population. Covariates entered the multivariable model if *p* < 0.10 at univariable analysis or based on clinical relevance; PlGF (dichotomized) was included a priori as the variable of primary interest. Continuous covariates were modeled on their native scale. To reduce the risk of overfitting given the available number of events, the primary multivariable model was restricted to four clinically relevant covariates (age, KPS, multifocality and PlGF). Alternative extended models are reported in the Supplementary Materials. Adjusted hazard ratios (HRs) with 95% confidence intervals (CIs) are reported; two-sided *p* < 0.05 was considered statistically significant. The same model specification was applied to the RPA 3–4 analysis. Kaplan–Meier curves were compared by the log-rank test.

No formal a priori sample size calculation was performed. This prospective exploratory cohort had a fixed accrual period, and the sample size was determined by the number of eligible patients. The primary endpoint was overall survival; effect sizes are reported with 95% CIs, and model complexity was restricted relative to the number of events to reduce overfitting.

## 3. Results

### 3.1. Demographic Characteristics

Fifty-four patients with IDH wild-type GBM undergoing surgery at the San Raffaele University Hospital (Milan) were included. There was a clear male predominance (male-to-female ratio 1.84:1). The median age was 63.8 years (Q1 = 51.9, Q3 = 68.8). By prognostic classes, 16 (32.7%), 29 (59.2%), and 4 (8.2%) patients were RPA 2, 3, and 4, respectively [7]. The median KPS at diagnosis was 80 (Q1 = 70, Q3 = 90). Thirty-six patients (66.7%) had some kind of comorbidity. Presenting symptoms were focal deficit in 33 (61.1%), seizures in 12 (22.2%), and cognitive impairment in 9 (16.7%) patients. At admission, 29 patients (58%) were receiving steroids, 16 (38.8%) antiepileptics, and 10 (18.9%) antiplatelet therapy. Additional baseline details are summarized in Table 1.

### 3.2. Pathological and Radiological Features

All tumors were IDH wild type. MGMT promoter methylation was present in 20 cases (40.8%). Median Ki67 and p53 expression were 25% (Q1 = 18, Q3 = 40) and 7% (Q1 = 1, Q3 = 30), respectively. Molecular and immunohistochemical features and imaging characteristics are summarized in Table 2.

Lesions were evenly distributed between hemispheres, with a slight left-sided predominance (29 cases; 53.7%). The median number of involved lobes was one (Q1 = 1, Q3 = 2). Radiological necrosis was observed in 48 patients (88.9%).

### 3.3. Quantification of Circulating Levels of Angiogenesis Mediators

Circulating levels of PlGF and other angiogenic mediators are reported in Table 3.

### 3.4. Correlations Between Key Angiogenesis Mediators

PlGF levels at diagnosis showed a strong correlation with FGF (ρ 0.710; *p* < 0.001) and moderate correlations with VEGF (ρ 0.491; *p* = 0.024), angiopoietin-1 (ρ 0.476; *p* < 0.001), IL-8 (ρ 0.581; *p* < 0.001), and TNF-α (ρ 0.491; *p* < 0.001). Significant correlations were also present with IL-6 (ρ 0.355; *p* < 0.001) and EGF (ρ 0.336; *p* = 0.013). The full correlation matrix is provided in Supplementary Table S1.

### 3.5. Adjuvant Therapies

Planned adjuvant regimens were: hypofractionated radiotherapy (HFRT) with sequential temozolomide (TMZ) in 2 patients (4.2%), standard radiotherapy (RT) with sequential TMZ in 3 patients (6.3%), HFRT with concomitant TMZ followed by sequential TMZ in 10 patients (20.8%), and RT with concomitant TMZ followed by sequential TMZ, in 31 patients (64.6%). Two patients (4.2%) received HFRT alone. Forty-three patients initiated sequential therapy, of whom 23 (53.5%) completed it. The median number of TMZ cycles was 5 (Q1 = 2, Q3 = 6). Nineteen patients (39.6%) discontinued therapy due to toxicity or progression, and in 6 (12.5%) sequential therapy was not planned because of early progression. Thirteen patients (27.1%) developed toxicity during therapy. Twenty-six patients (61.9%) received second-line therapies: regorafenib (*n* = 13), TMZ (*n* = 10), bevacizumab (*n* = 6), and lomustine (*n* = 5). Complete treatment data are reported in Supplementary Table S5.

### 3.6. Survival

Median overall survival (OS) and progression-free survival (PFS) were 17.7 months (95% CI 12.1–23.2) and 8.8 months (95% CI 6.9–10.8), respectively. Survival estimates are summarized in Supplementary Table S6; Kaplan-Meier curves for OS and PFS in the whole cohort are shown in Supplementary Figure S1.

**Figure 1:**
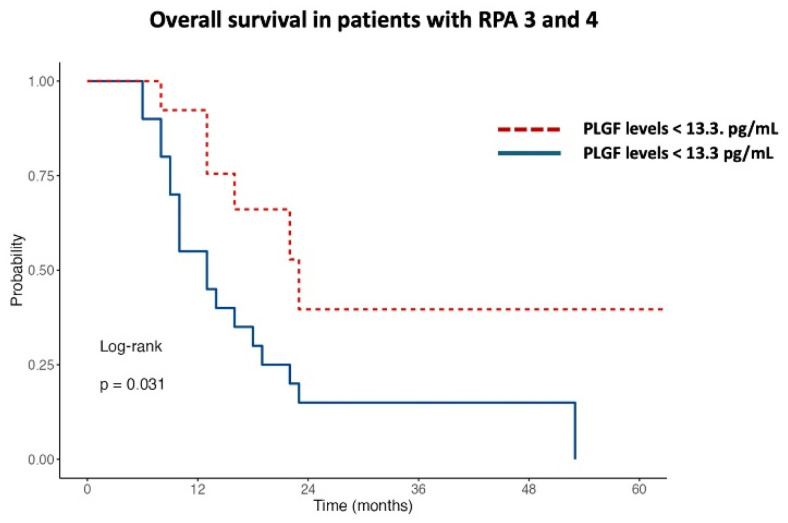
Kaplan Meier curves depicting overall survival in high-risk patients (RPA 3 and 4) stratified according to circulating PlGF levels.

In patients with RPA 3–4 (*n* = 33), median OS and PFS were 16.3 months (95% CI 11.0–21.5) and 7.2 months (95% CI 6.0–8.4), respectively. The OS cut-off for PlGF derived by the maximally selected rank statistics was 13.3 pg/mL. Kaplan-Meier analysis showed longer OS in the low-PlGF group. This difference became statistically significant when focusing on high-risk patients (RPA 3–4), where low PlGF identified a subgroup with markedly improved survival (median OS 17.6 vs. 8.5 months; *p* = 0.031), as shown in Figure 1.

**Figure 2:**
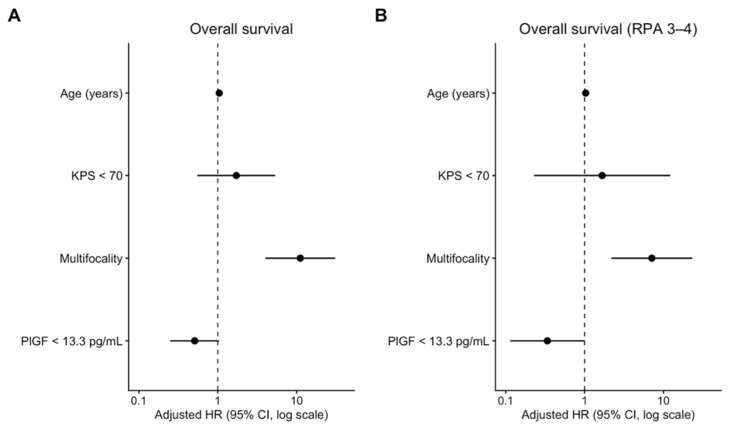
Forest plots of the reduced multivariable Cox regression models for overall survival. (**A**) Overall cohort. (**B**) RPA 3–4 subgroup. Points represent adjusted hazard ratios (HRs); horizontal lines indicate 95% confidence intervals. The dashed vertical line indicates HR = 1. Models were adjusted for age, Karnofsky Performance Status, multifocality, and baseline PlGF concentration (<13.3 pg/mL).

In the overall cohort, multifocality was independently associated with shorter OS (HR = 11.119, 95% CI 4.015–30.792; *p* < 0.001), and age remained adverse (per year: HR = 1.043, 95% CI 1.004–1.084; *p* = 0.030). KPS < 70 was not significantly associated with OS (HR = 1.718, 95% CI 0.552–5.342; *p* = 0.350). PlGF < 13.3 pg/mL showed a protective trend that did not reach statistical significance in the overall cohort (HR = 0.510, 95% CI 0.250–1.039; *p* = 0.064); see Figure 2A and Table 4.

In the RPA 3–4 subgroup, multifocality remained strongly associated with worse OS (HR = 7.110, 95% CI 2.179–23.201; *p* = 0.001), whereas age (HR = 1, 95% CI 0.975–1.094; *p* = 0.277) and KPS < 70 (HR = 1.669, 95% CI 0.228–12.197; *p* = 0.614) were not significant. PlGF < 13.3 pg/mL identified patients with a lower hazard of death, retaining a protective association in the reduced multivariable model (HR = 0.337, 95% CI 0.114–0.999; *p* = 0.050); see Figure 2B and Table 4. The protective trend of low PlGF values was confirmed in alternative Cox regression models including additional exploratory covariates and a sensitivity analysis adjusting for dexamethasone dosage (Supplementary Tables S3A,B and S4). Univariate Cox regression results are reported in Supplementary Table S2.

Longitudinally, PlGF increased at recurrence in patients with EGFR amplification (*p* = 0.062), TERT mutation (*p* = 0.042), TMZ toxicity (*p* = 0.017), or lomustine treatment (*p* = 0.050); see Supplementary Figure S2.

## 4. Discussion

Angiogenesis is a multifactorial mechanism that supports and promotes glioblastoma (GBM) growth and progression. Recent evidence highlights its central role in the development of resistance to adjuvant therapies, particularly temozolomide (TMZ) chemoresistance [2,10,11,12]. The biological process encompasses vasogenesis and vascular permeability, immunomodulation toward onco-permissive phenotypes, and tissue remodeling (epithelial-to-mesenchymal transition, EMT). These mechanisms are interdependent and share predominantly autocrine and paracrine mediators, including cytokines, growth factors, transcription factors, membrane proteins, and interleukins. Ultimately, blood–brain barrier (BBB) permeabilization has led to the hypothesis that these molecules exert endocrine effects; studies quantifying the systemic levels of these mediators have recently been published [13].

Many angiogenic factors have been characterized by leveraging knowledge from other diseases. In the last decade, interest has grown around placental growth factor (PlGF), a key player in BBB permeability in eclampsia [4], with roles described in other cancers involving angiogenesis [6], progression [14], and stemness [15]. Evidence in GBM remains limited. In general, PlGF has been observed to vary with intracranial tumor type, with specificity reported for GBM and brain metastases, while transcript levels do not appear to correlate with tumor grade [16].

Current knowledge suggests that PlGF primarily acts in synergy with VEGF through VEGFR-1 signaling [17,18], contributing to angiogenesis and myeloid-mediated immunomodulation [19,20]. Preclinical studies support its role in tumor invasiveness and vascular remodeling, and interactions with VEGF pathways have been described in glioblastoma [21,22]. Consistent with this framework, the present study confirmed a correlation between circulating PlGF and VEGF (*p* = 0.024).

From a pathophysiological standpoint, PlGF has been implicated in inefficient vasculature characterized by a high cerebral blood flow/volume ratio and increased vessel caliber [17]; GBM cancer stem-cell xenografts overexpressing PlGF exhibited increased vascular diameter [23]. In vitro studies using brain endothelial and glioblastoma-derived endothelial cells indicated that PlGF and VEGF are co-regulated by microenvironmental factors such as hypoxia and growth factor signaling, contributing to vascular remodeling and tumor progression [17,24,25].

The role of bevacizumab on PlGF levels is debated. Immunohistochemistry has shown PlGF reduction after bevacizumab [17] and increased levels in refractory patients [26], while other evidence questions PlGF’s role in resistance to anti-angiogenic therapy [27]. This has motivated combinations of bevacizumab with inhibitors targeting PlGF/VEGF or their receptors [28,29,30]. In our cohort, we did not observe significant differences in circulating PlGF between diagnosis and recurrence overall, including among patients treated with bevacizumab. These findings should be interpreted with caution given the limited number and heterogeneity of bevacizumab-treated patients.

To our knowledge, this study provides the first characterization of systemic PlGF and its interaction with established angiogenic mediators in relation to clinical and oncological outcomes in GBM. Interest in circulating biomarkers and liquid biopsy approaches in neuro-oncology has recently expanded, highlighting the potential clinical value of blood-based biomarkers for diagnosis, prognostic stratification, and disease monitoring [31].

Fifty-four IDH-wildtype patients underwent blood sampling at diagnosis and recurrence and were prospectively enrolled, with a median follow-up of 16.2 months (Q1 = 12.4, Q3 = 23.2). We correlated plasma levels within a 10-analyte angiogenesis panel with demographic, clinical, radiological, pathological, and treatment-related variables.

We observed significant correlations between PlGF and multiple angiogenic and inflammatory mediators, including IL-6, angiopoietin-1, IL-8, and TNF-a, consistent with its role within a complex pro-angiogenic network. Notably, the strong association with IL-6 (*p* < 0.001) represents an interesting observation in GBM and supports a link with neuroinflammatory and blood–brain barrier-related processes [4]. We also confirmed associations with angiopoietin-1 (*p* < 0.001), IL-8 (*p* < 0.001), and TNF-α (*p* < 0.001), in line with PlGF involvement in vascular remodeling and pro-angiogenic inflammatory signaling [5,32,33,34]. In neuro-oncology, the mutual variations of these values have been studied in relation to bevacizumab administration [26]. The observed relationships with EGF (*p* = 0.013) and FGF (*p* < 0.001) further support the integration of PlGF with broader angiogenic signaling networks [35,36]. However, these findings should be interpreted as descriptive associations rather than evidence of direct mechanistic interactions. Further experimental studies are warranted to clarify the biological mechanisms underlying these relationships.

Tumor analysis showed relationships between baseline circulating PlGF, Ki67, and p53 expression (*p* = 0.008 and *p* = 0.046, respectively). The association with p53 was preclinically anticipated in gastric cancer: in PlGF-knockdown mice, survival and tumor migration were inhibited through p53-related mechanisms [37]. We also observed an inverse correlation between PlGF and EGFR amplification (*p* < 0.001) and an association with ATRX mutation (*p* < 0.001). To our knowledge, the latter has been only scarcely investigated in GBM, while data on EGFR-PlGF interactions are scarce and largely preclinical from an in vivo model of metastatic triple-negative breast cancer [38]. Regarding neuroimaging, the FLAIR/contrast enhancement ratio—an indirect index of vascular permeability—correlated with PlGF (*p* = 0.003), in line with prior immunohistochemical observations [26]. From a clinical standpoint, higher PlGF was associated with peripheral arterial and hematological disease (*p* < 0.001, and *p* = 0.002, respectively), and the use of cardioaspirin (*p* = 0.026), consistent with PlGF’s role in cerebrovascular disease [39] and reports of cardioaspirin-induced apoptosis via STAT3 blockade in GBM cells [40]. No associations emerged with steroid or anti-epileptic therapy. Overall, these findings suggest that circulating PlGF may reflect specific molecular features of glioblastoma biology. However, given the exploratory nature of these analyses and the limited sample size, these associations should be interpreted cautiously and require validation in larger molecularly characterized cohorts.

At recurrence, unlike a prior RNA-based study that reported decreased PlGF expression in tumor tissue [41], we did not detect significant changes in circulating levels overall, noting the difference in biological material (tissue vs. plasma).

Finally, although some studies report limited prognostic utility for individual angiogenic markers [42,43], we observed that low baseline PlGF identified a subgroup with longer survival among high-risk patients (RPA 3–4; log rank *p* = 0.031, Figure 1) and remained protective in multivariable models (Table 4 and Figure 2). In the overall cohort, the association did not reach statistical significance. Overall, these findings suggest that the prognostic value of PlGF becomes more evident when considering a clinically high-risk population rather than the entire heterogeneous cohort. The robustness of the association was further supported by alternative multivariable models, including extended models and a sensitivity analysis adjusting for corticosteroid exposure (Supplementary Tables S3A and S4). The subgroup analysis was predefined based on the established prognostic role of the RPA classification and our hypothesis that PlGF might provide additional prognostic information in clinically high-risk patients. Accordingly, the prognostic relevance of circulating PlGF should currently be interpreted as being primarily supported in this predefined high-risk subgroup rather than in the overall GBM population.

As anticipated, a comparative analysis found no significant differences in PlGF levels at diagnosis and recurrence. However, exploratory longitudinal analysis suggested an increase in PlGF at recurrence in selected molecular and treatment-defined subgroups (Supplementary Figure S2). Given the limited number of paired samples available for these analyses, these findings should be interpreted with caution and considered hypothesis-generating.

### Limitations

This study has some limitations. First, the relatively limited sample size may have reduced statistical power, particularly for subgroup and multivariable analyses. Therefore, the present findings should be considered exploratory and hypothesis-generating, requiring validation in larger independent prospective cohorts before clinical implementation. Second, the optimal PlGF cutoff was derived from the present dataset using maximally selected rank statistics. Although this approach is widely used in exploratory biomarker studies, it may overestimate the prognostic performance of the selected threshold. Accordingly, the proposed cutoff requires independent external validation before clinical application. Third, as with any exploratory biomarker study involving multiple subgroup and correlation analyses, the possibility of type I error cannot be excluded. The reported associations should therefore be interpreted cautiously until independently validated. Fourth, although no significant association between baseline circulating PlGF levels and corticosteroid or anti-epileptic therapy was observed, treatment heterogeneity, concomitant medications, and patient comorbidities may still have influenced circulating biomarker levels. In addition, the longitudinal analyses at recurrence were performed in a limited number of paired samples and should be considered exploratory. Finally, baseline circulating PlGF was measured at a single pre-treatment time point, which reflects its intended use as a baseline prognostic biomarker but does not capture intra-patient variability or temporal changes during disease evolution and treatment.

## 5. Conclusions

Placental growth factor (PlGF) is a circulating mediator involved in angiogenesis in glioblastoma, reflecting both angiogenic signaling and radiopathological features of tumor biology.

In high-risk patients (RPA 3–4), low baseline PlGF levels identify a subgroup with longer overall survival and retain a protective association in multivariable models, while a weaker effect is observed in the overall cohort. Exploratory analyses suggest that PlGF may increase at recurrence in selected molecular and treatment-defined subgroups. No association with bevacizumab exposure was observed, suggesting involvement of alternative angiogenic pathways.

These findings support the potential role of circulating PlGF as a surrogate marker of the neurovascular microenvironment and a promising candidate prognostic biomarker in high-risk GBM patients. However, given the exploratory nature of the present study, the limited sample size, and the lack of external validation, larger prospective studies are required before considering the clinical implementation of circulating PlGF or its incorporation into biomarker-driven anti-angiogenic strategies.

## Figures and Tables

**Table 1:** Baseline clinical and demographic characteristics.

Characteristic	Overall	N
Age, years—median (Q1–Q3)	63.8 (51.9–68.8)	54
Male sex—*n* (%)	35 (64.8) male, 19 (35.2) female	54
KPS—median (Q1–Q3)	80 (70–90)	54
RPA class—*n* (%)	1: 0 (0) 2: 16 (32.7) 3: 29 (59.2) 4: 4 (8.2)	49
Comorbidities—*n* (%)	Total: 36 (66.7) Hypertension: 20 (37.0) Cognitive deterioration: 6 (11.1) Chronic Obstructive Pulmonary Disease: 5 (9.3) Thyropathy: 5 (9.3) History of other neoplasm: 5 (9.3%) Diabetes: 4 (7.4) Ischemic cardiovascular disease: 3 (5.6) Hepatopathy: 3 (5.6) Autoimmune disease: 3 (5.6) Hematologic disease: 3 (5.6) Benign prostatic hyperplasia: 1 (1.9) Atrial fibrillation: 1 (1.9) Peripheral artery disease: 1 (1.9) Deep vein thrombosis: 1 (1.9)	54
Presenting symptoms—*n* (%)	Seizures: 12 (22.2) Focal deficit: 33 (61.1) Cognitive decline: 9 (16.7)	54
Steroids at admission—*n* (%)	29 (58.0)	50
Steroids dose/weight—median (Q1–Q3)	0.125 (0.1–0.182)	29
Antiepileptics—*n* (%)	Total: 16 (30.8) Levetiracetam: 14 (87.5) Lacosamide: 1 (6.25) Carbamazepine: 1 (6.25)	52
Antiplatelet drugs—*n* (%)	Total: 10 (18.9) Aspirin: 8 (80.0) Clopidogrel: 2 (20.0)	53
Extent of resection—*n* (%)	Gross total: 31 (57.4) Subtotal: 23 (42.6)	54

Table legend. The number of observations for each variable is indicated in the last column. For categorical variables, absolute numbers and percentages (in brackets) are indicated. KPS = Karnofsky Performance Status. RPA classification was available for 49 of 54 patients. Percentages were calculated using patients with available RPA classification (*n* = 49).

**Table 2:** Histological, molecular, and radiological characterization.

Variable	Overall	N
Pathology/Molecular		
IDH status—wild-type, *n* (%)	54 (100)	54
MGMT promoter methylation—*n* (%)	20 (40.8)	49
Ki-67 expression %—median (Q1–Q3)	25 (18–40)	53
p53 expression %—median (Q1–Q3)	7 (1–30)	53
EGFR amplification—*n* (%)	9 (81.8)	11
TERT mutation	6 (75)	8
ATRX loss of expression—*n* (%)	3 (6.0)	50
Radiology and volumes		
Hemisphere—side, *n* (%)	Right 25 (46.3) Left 29 (53.7)	52
Deep location—*n* (%)	12 (22.2)	52
Multifocal disease—*n* (%)	7 (13)	52
Midline shift—*n* (%)	20 (37.7)	52
Necrosis on MRI—*n* (%)	48 (92.3)	52
FLAIR volume, cc—median (Q1–Q3)	92.2 (50.9–137)	52
CE volume, cc—median (Q1–Q3)	23.3 (12.9–36)	52
Necrosis (N) volume, cc—median (Q1–Q3)	5.56 (1.82–12.4)	52
FLAIR minus CE volume, cc—median (Q1–Q3)	64.4 (29.9–106)	52
CE minus N volume, cc—median (Q1–Q3)	15.5 (9.55–23.1)	52
FLAIR/CE volume ratio—median (Q1–Q3)	3.74 (2.11–5.46)	52

Table legend. The number of observations for each variable is indicated in the last column. For categorical variables, absolute numbers and percentages (in brackets) are indicated. CE = contrast-enhancement.

**Table 3:** Quantification of circulating levels of PLGF and angiogenic mediators.

Analyte (pg/mL)	Median (Q1–Q3)
PlGF	6.88 (3.52–18.3)
IL-6	2.75 (0.0–5.02)
Angiopoietin-1	32,046 (18,018–49,058)
Angiopoietin-2	5027 (2944–6373)
EGF	312 (171–559)
FGF	835 (318–1485)
IL-8	25 (16.9–43)
PECAM-1	19,915 (14,569–31,144)
VEGF	332 (201–391)
TNF-α	21.7 (13.2–46.8)

Table legend. PlGF = placental growth factor; EGF = epidermal growth factor; FGF = fibroblast growth factor; PECAM-1 = platelet endothelial cell adhesion molecule; VEGF = vascular endothelial growth factor; TNF-α = tumor necrosis factor α.

**Table 4:** Multivariable survival analysis.

Variable	Overall Cohort (*n* = 54)			RPA 3–4 (*n* = 33)		
	HR	95% CI	*p* Value	HR	95% CI	*p* Value
Age (years)	1.043	1.004–1.084	0.030 *	1.033	0.975–1.094	0.277
KPS < 70 vs. ≥70	1.718	0.552–5.342	0.350	1.669	0.228–12.197	0.614
Multifocality	11.119	4.015–30.792	<0.001 **	7.110	2.179–23.201	0.001 **
PlGF < 13.3 pg/mL	0.510	0.250–1.039	**0.064**	0.337	0.114–0.999	0.050 *

Table legend. Multivariable survival analysis in the total population and patients with RPA = 3–4. KPS = Karnofsky Performance Status; HR = Hazard Ratio; RPA = Recursive Partitioning Analysis. **Bold** indicates statistically significant results. * *p* < 0.050. ** Statistically significant in both the overall cohort and the RPA 3–4 subgroup.

## Data Availability

De-identified clinical data, plasma assay data, and analysis code are available from the corresponding author upon reasonable request, subject to institutional and ethical restrictions related to patient privacy.

## Supplementary Materials

The following supporting information can be downloaded at: https://www.mdpi.com/article/10.3390/biomedicines14071628/s1, Supplementary Figure S1. Kaplan Meier curves depicting overall and progression-free survival in the whole population. Supplementary Figure S2. Box-plot depicting PlGF levels at baseline and relapse in se-lected cohort of patients. Abbreviations: CT = chemotherapy. Supplementary Table S1. Spearman’s correlations among key angiogenetic cytokines. Supplementary Table S2. Univariate Cox regression analysis for overall survival in the overall cohort. Supplementary Table S3A. Multivariable survival analysis, extended model. Supplementary Table S3B. Multivariable survival analysis, extended and alternative model. Supplementary Table S4. Alternative multivariable Cox regression models with steroid therapy as a covariate. Supplementary Table S5. Adjuvant therapies, toxicity and therapeutic compliance. Supplementary Table S6. Survival estimates.

## Abbreviations

ANOVA, Analysis of Variance; BBB, Blood–brain Barrier; CE, Contrast Enhancement; CI, Confidence Interval; EMT, Epithelial-to-Mesenchymal Transition; EGF, Epidermal Growth Factor; EOR, Extent of Resection; FGF, Fibroblast Growth Factor; FLAIR, Fluid-Attenuated Inversion Recovery; GBM, Glioblastoma; HFRT, Hypofractionated Radiotherapy; HR, Hazard Ratio; IDH, Isocitrate Dehydrogenase; IL, Interleukin; IQR, Interquartile Range; KPS, Karnofsky Performance Status; MRI, Magnetic Resonance Imaging; OS, Overall Survival; PFS, Progression-Free Survival; PlGF, Placental Growth Factor; RANO, Response Assessment in Neuro-Oncology; RPA, Recursive Partitioning Analysis; RT, Radiotherapy; SD, Standard Deviation; TMZ, Temozolomide; TNF-α, Tumor Necrosis Factor Alpha; VEGF, Vascular Endothelial Growth Factor.

## References

[1] Zabihi A. (2025). The role of biological macromolecules in the regulation of angiogenesis in glioblastoma: Focus on vascular growth factors, integrins, and extracellular matrix proteins. Int. J. Biol. Macromol..

[2] Lv G., Li X., Deng H., Zhang J., Gao X. (2024). Regulatory Mechanisms of STAT3 in GBM and its Impact on TMZ Resistance. Curr. Mol. Pharmacol..

[3] Kim S., Kim K.H., Jung H.W., Jeong E.O., Lee H.J., Kwon J., Kwon H.J., Choi S.W., Koh H.S., Kim S.H. (2025). Elevated Serum IL-6 as a Negative Prognostic Biomarker in Glioblastoma: Integrating Bioinformatics and Clinical Validation. J. Cancer.

[4] Bucher V., Herrock O.T., Schell S., Visser J., Imberg H., Burke J., Zetterberg H., Blennow K., Walker S.P., Tong S. (2025). Blood-brain barrier injury and neuroinflammation in pre-eclampsia and eclampsia. eBioMedicine.

[5] Huang Z., Chen L.J., Huang D., Yi J., Chen Z., Lin P., Wang Y., Zheng J., Chen W. (2024). Preoperative Intravitreal Conbercept Injection Reduced Both Angiogenic and Inflammatory Cytokines in Patients with Proliferative Diabetic Retinopathy. J. Diabetes Res..

[6] Aktas S.H., Akbulut Yazici H.O., Zengin N., Akgun H.N., Ustuner Z., Icli F. (2017). A new angiogenesis prognostic index with VEGFA, PlGF, and angiopoietin1 predicts survival in patients with advanced gastric cancer. Turk. J. Med. Sci..

[7] Park Y.W., Choi K.S., Foltyn-Dumitru M., Brugnara G., Banan R., Kim S., Han K., Park J.E., Kessler T., Bendszus M. (2024). Incorporating Supramaximal Resection into Survival Stratification of IDH-wildtype Glioblastoma: A Refined Multi-institutional Recursive Partitioning Analysis. Clin. Cancer Res..

[8] Curran W.J., Scott C.B., Horton J., Nelson J.S., Weinstein A.S., Fischbach A.J., Chang C.H., Rotman M., Asbell S.O., Krisch R.E. (1993). Recursive partitioning analysis of prognostic factors in three Radiation Therapy Oncology Group malignant glioma trials. J. Natl. Cancer Inst..

[9] Wen P.Y., Macdonald D.R., Reardon D.A., Cloughesy T.F., Sorensen A.G., Galanis E., Degroot J., Wick W., Gilbert M.R., Lassman A.B. (2010). Updated response assessment criteria for high-grade gliomas: Response assessment in neuro-oncology working group. J. Clin. Oncol..

[10] Beylerli O., Gareev I., Musaev E., Ilyasova T., Roumiantsev S., Chekhonin V. (2025). Angiogenesis and Resistance Mechanisms in Glioblastoma: Targeting Alternative Vascularization Pathways to Overcome Therapy Resistance. Curr. Pharm. Des..

[11] Bayona C., Randelovic T., Ochoa I. (2026). Tumor microenvironment in glioblastoma: The central role of the hypoxic-necrotic core. Cancer Lett..

[12] Ballato M., Germana E., Ricciardi G., Giordano W.G., Tralongo P., Buccarelli M., Castellani G., Ricci-Vitiani L., D’Alessandris Q.G., Giuffre G. (2025). Understanding Neovascularization in Glioblastoma: Insights from the Current Literature. Int. J. Mol. Sci..

[13] Bender D.E., Schaettler M.O., Sheehan K.C., Johanns T.M., Dunn G.P. (2021). Cytokine Profiling in Plasma from Patients with Brain Tumors Versus Healthy Individuals using 2 Different Multiplex Immunoassay Platforms. Biomark. Insights.

[14] Albonici L., Giganti M.G., Modesti A., Manzari V., Bei R. (2019). Multifaceted Role of the Placental Growth Factor (PlGF) in the Antitumor Immune Response and Cancer Progression. Int. J. Mol. Sci..

[15] Mahmoodi F., Akrami H. (2017). PlGF Knockdown Decreases Tumorigenicity and Stemness Properties of Spheroid Body Cells Derived from Gastric Cancer Cells. J. Cell. Biochem..

[16] Ilhan-Mutlu A., Wagner L., Widhalm G., Wohrer A., Bartsch S., Czech T., Heinzl H., Leutmezer F., Prayer D., Marosi C. (2013). Exploratory investigation of eight circulating plasma markers in brain tumor patients. Neurosurg. Rev..

[17] Gerstner E.R., Emblem K.E., Yen Y.F., Dietrich J., Jordan J.T., Catana C., Wenchin K.L., Hooker J.M., Duda D.G., Rosen B.R. (2020). Vascular dysfunction promotes regional hypoxia after bevacizumab therapy in recurrent glioblastoma patients. Neurooncol. Adv..

[18] Luttun A., Autiero M., Tjwa M., Carmeliet P. (2004). Genetic dissection of tumor angiogenesis: Are PlGF and VEGFR-1 novel anti-cancer targets?. Biochim. Biophys. Acta.

[19] Lisi L., Pia Ciotti G.M., Chiavari M., Ruffini F., Lacal P.M., Graziani G., Navarra P. (2020). Vascular endothelial growth factor receptor 1 in glioblastoma-associated microglia/macrophages. Oncol. Rep..

[20] Thomas A.A., Fisher J.L., Hampton T.H., Christensen B.C., Tsongalis G.J., Rahme G.J., Whipple C.A., Steel S.E., Davis M.C., Gaur A.B. (2017). Immune modulation associated with vascular endothelial growth factor (VEGF) blockade in patients with glioblastoma. Cancer Immunol. Immunother..

[21] Atzori M.G., Tentori L., Ruffini F., Ceci C., Lisi L., Bonanno E., Scimeca M., Eskilsson E., Daubon T., Miletic H. (2017). The anti-vascular endothelial growth factor receptor-1 monoclonal antibody D16F7 inhibits invasiveness of human glioblastoma and glioblastoma stem cells. J. Exp. Clin. Cancer Res..

[22] Szabo E., Schneider H., Seystahl K., Rushing E.J., Herting F., Weidner K.M., Weller M. (2016). Autocrine VEGFR1 and VEGFR2 signaling promotes survival in human glioblastoma models in vitro and in vivo. Neuro Oncol..

[23] Xu L., Cochran D.M., Tong R.T., Winkler F., Kashiwagi S., Jain R.K., Fukumura D. (2006). Placenta growth factor overexpression inhibits tumor growth, angiogenesis, and metastasis by depleting vascular endothelial growth factor homodimers in orthotopic mouse models. Cancer Res..

[24] Krishnan S., Szabo E., Burghardt I., Frei K., Tabatabai G., Weller M. (2015). Modulation of cerebral endothelial cell function by TGF-beta in glioblastoma: VEGF-dependent angiogenesis versus endothelial mesenchymal transition. Oncotarget.

[25] Chedeville A.L., Lourdusamy A., Monteiro A.R., Hill R., Madureira P.A. (2020). Investigating Glioblastoma Response to Hypoxia. Biomedicines.

[26] Ezaki T., Tanaka T., Tamura R., Ohara K., Yamamoto Y., Takei J., Morimoto Y., Imai R., Kuranai Y., Akasaki Y. (2024). Status of alternative angiogenic pathways in glioblastoma resected under and after bevacizumab treatment. Brain Tumor Pathol..

[27] Schneider K., Weyerbrock A., Doostkam S., Plate K., Machein M.R. (2015). Lack of evidence for PlGF mediating the tumor resistance after anti-angiogenic therapy in malignant gliomas. J. Neurooncol..

[28] de Groot J.F., Piao Y., Tran H., Gilbert M., Wu H.K., Liu J., Bekele B.N., Cloughesy T., Mehta M., Robins H.I. (2011). Myeloid biomarkers associated with glioblastoma response to anti-VEGF therapy with aflibercept. Clin. Cancer Res..

[29] Lassen U., Chinot O.L., McBain C., Mau-Sorensen M., Larsen V.A., Barrie M., Roth P., Krieter O., Wang K., Habben K. (2015). Phase 1 dose-escalation study of the antiplacental growth factor monoclonal antibody RO5323441 combined with bevacizumab in patients with recurrent glioblastoma. Neuro Oncol..

[30] Gerstner E.R., Eichler A.F., Plotkin S.R., Drappatz J., Doyle C.L., Xu L., Duda D.G., Wen P.Y., Jain R.K., Batchelor T.T. (2011). Phase I trial with biomarker studies of vatalanib (PTK787) in patients with newly diagnosed glioblastoma treated with enzyme inducing anti-epileptic drugs and standard radiation and temozolomide. J. Neurooncol..

[31] Ruda R., Pellerino A., Soffietti R. (2024). Blood and cerebrospinal fluid biomarkers in neuro-oncology. Curr. Opin. Neurol..

[32] Machado J.S.R., Machado M.S.R., Bertagnolli T.V., Martins L.A.B., Freitas S.F., Ovidio P.P., Sandrim V.C., Cardoso V.C., Bettiol H., Barbieri M.A. (2019). Role of plasma PlGF, PDGF-AA, ANG-1, ANG-2, and the ANG-1/ANG-2 ratio as predictors of preeclampsia in a cohort of pregnant women. Pregnancy Hypertens..

[33] Grodecka-Szwajkiewicz D., Ulanczyk Z., Zagrodnik E., Luczkowska K., Roginska D., Kawa M.P., Stecewicz I., Safranow K., Machalinski B. (2020). Differential Secretion of Angiopoietic Factors and Expression of MicroRNA in Umbilical Cord Blood from Healthy Appropriate-For-Gestational-Age Preterm and Term Newborns-in Search of Biomarkers of Angiogenesis-Related Processes in Preterm Birth. Int. J. Mol. Sci..

[34] Simon T., Coquerel B., Petit A., Kassim Y., Demange E., Le Cerf D., Perrot V., Vannier J.P. (2014). Direct effect of bevacizumab on glioblastoma cell lines in vitro. Neuromol. Med..

[35] Afrin S., El Sabah M., Manzoor A., Miyashita-Ishiwata M., Reschke L., Borahay M.A. (2023). Adipocyte coculture induces a pro-inflammatory, fibrotic, angiogenic, and proliferative microenvironment in uterine leiomyoma cells. Biochim. Biophys. Acta Mol. Basis Dis..

[36] Cazzato G., Ingravallo G., Ribatti D. (2024). Angiogenesis Still Plays a Crucial Role in Human Melanoma Progression. Cancers.

[37] Akrami H., Mahmoodi F., Havasi S., Sharifi A. (2016). PlGF knockdown inhibited tumor survival and migration in gastric cancer cell via PI3K/Akt and p38MAPK pathways. Cell Biochem. Funct..

[38] Roberti M.P., Arriaga J.M., Bianchini M., Quinta H.R., Bravo A.I., Levy E.M., Mordoh J., Barrio M.M. (2012). Protein expression changes during human triple negative breast cancer cell line progression to lymph node metastasis in a xenografted model in nude mice. Cancer Biol. Ther..

[39] Che P., Wang S., Liu X., Lu Y., Tian Z., Feng Q., Chen F., Zhang N. (2025). Plasma placental growth factor as a biomarker for subcortical ischemic vascular dementia and its cognitive correlation mediated by white matter hyperintensities. Alzheimers Dement..

[40] Kim S.R., Bae M.K., Kim J.Y., Wee H.J., Yoo M.A., Bae S.K. (2009). Aspirin induces apoptosis through the blockade of IL-6-STAT3 signaling pathway in human glioblastoma A172 cells. Biochem. Biophys. Res. Commun..

[41] Tabouret E., Denicolai E., Delfino C., Graillon T., Boucard C., Nanni I., Padovani L., Figarella-Branger D., Chinot O. (2016). Changes in PlGF and MET-HGF expressions in paired initial and recurrent glioblastoma. J. Neurooncol..

[42] Labussiere M., Cheneau C., Prahst C., Gallego Perez-Larraya J., Farina P., Lombardi G., Mokhtari K., Rahimian A., Delattre J.Y., Eichmann A. (2016). Angiopoietin-2 May Be Involved in the Resistance to Bevacizumab in Recurrent Glioblastoma. Cancer Investig..

[43] Nayak L., Molinaro A.M., Peters K., Clarke J.L., Jordan J.T., de Groot J., Nghiemphu L., Kaley T., Colman H., McCluskey C. (2021). Randomized Phase II and Biomarker Study of Pembrolizumab plus Bevacizumab versus Pembrolizumab Alone for Patients with Recurrent Glioblastoma. Clin. Cancer Res..

