# Supplementary material for: Circulating Placental Growth Factor as a Prognostic Biomarker in High-Risk Glioblastoma Patients
Source: Biomedicines. 2026 Jul 20;14(7):1628. doi: 10.3390/biomedicines14071628 (PMC13407414; doi:10.3390/biomedicines14071628)
Supplement: Supplementary file 1 [file biomedicines-14-01628-s001.zip › biomedicines-4384923-supplementary.pdf]

Supplementary Figure S1

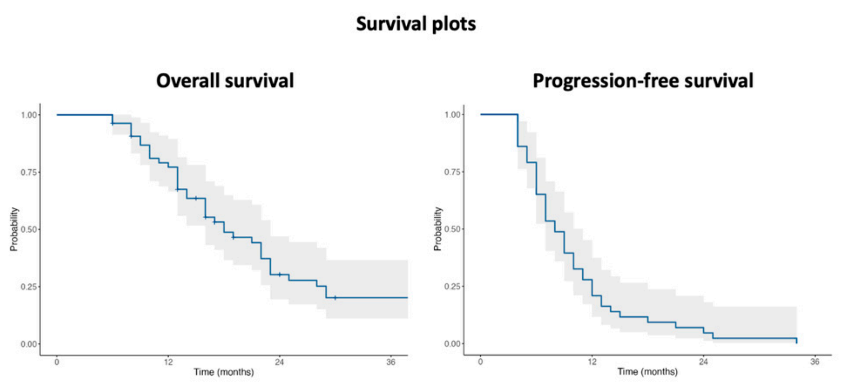

Figure legend. Kaplan Meier curves depicting overall and progression-free survival in the whole population.

Supplementary Figure S2

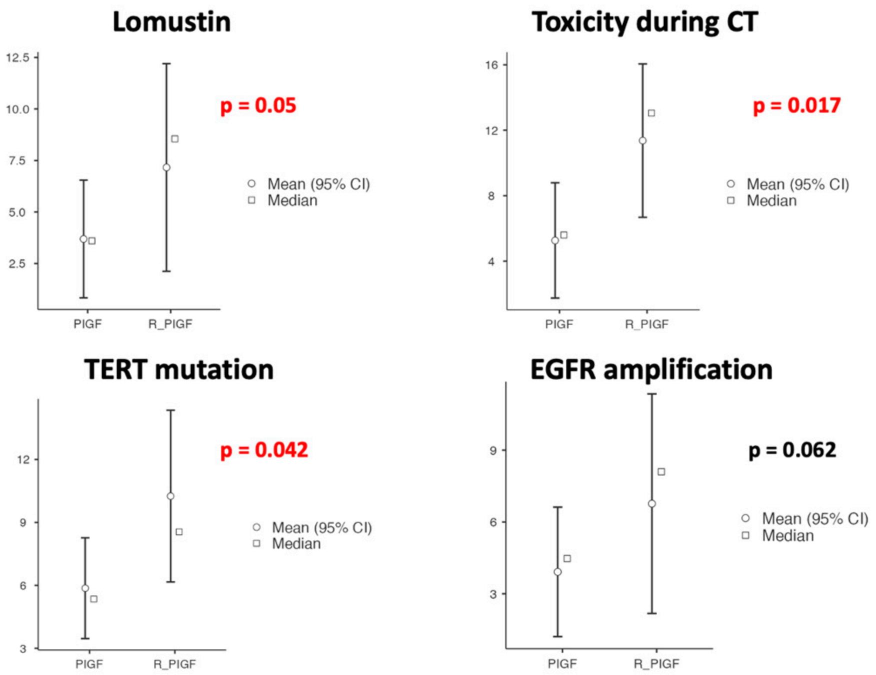

Figure legend. Box-plot depicting PlGF levels at baseline and relapse in selected cohort of patients. Abbreviations: CT = chemotherapy.

Table S1. Spearman's correlation among key angiogenetic cytokines.

	**PlGF**	**IL-6**	**Angiopoietin-1**	**Angiopoietin-2**	**EGF**	**FGF**	**IL-8**	**PECAM-1**	**VEGF**	**TNF-α**	**P-selectin**
**PlGF**	-										
**IL-6**	**ρ 0.355<br>p <.001****	-									
**Angiopoietin-1**	**ρ 0.476<br>p <.001****	ρ 0.228<br>p = 0.097	-								
**Angiopoietin-2**	ρ 0.162<br>p = 0.242	ρ 0.359<br>p = 0.008**	ρ 0.070<br>p = 0.614	-							
**EGF**	**ρ 0.336<br>p = 0.013***	ρ 0.021<br>p = 0.880	ρ 0.617<br>p <.001**	ρ -0.071<br>p = 0.607	-						
**FGF**	**ρ 0.710<br>p <.001****	ρ 0.223<br>p = 0.106	ρ 0.331<br>p = 0.015*	ρ 0.116<br>p = 0.403	ρ 0.377<br>p = 0.005**	-					
**IL-8**	**ρ 0.581<br>p <.001****	ρ 0.479<br>p <.001**	ρ 0.309<br>p = 0.023*	ρ 0.105<br>p = 0.449	ρ 0.316<br>p = 0.020*	ρ 0.447<br>p <.001**	-				
**PECAM-1**	ρ 0.145<br>p = 0.296	ρ -0.058<br>p = 0.675	ρ 0.176<br>p = 0.204	ρ 0.218<br>p = 0.112	ρ 0.197<br>p = 0.153	ρ -0.052<br>p = 0.711	ρ 0.026<br>p = 0.852	-			
**VEGF**	**ρ 0.307<br>p = 0.024***	ρ 0.185<br>p = 0.181	ρ 0.380<br>p = 0.005**	ρ 0.234<br>p = 0.089	ρ 0.438<br>p = 0.001**	ρ 0.291<br>p = 0.032*	ρ 0.378<br>p = 0.005**	ρ 0.241<br>p = 0.079	-		
**TNF-α**	**ρ 0.491<br>p <.001****	ρ 0.326<br>p = 0.016*	ρ 0.286<br>p = 0.036*	ρ -0.072<br>p = 0.605	ρ 0.238<br>p = 0.083	ρ 0.255<br>p <.001**	ρ 0.482<br>p <.001**	ρ -0.220<br>p = 0.111	ρ 0.233<br>p = 0.090	-	
**P-selectin**	ρ 0.004<br>p = 0.980	ρ 0.241<br>p = 0.085	ρ 0.166<br>p = 0.240	ρ 0.215<br>p = 0.125	ρ -0.034<br>p = 0.812	ρ -0.054<br>p = 0.706	ρ 0.199<br>p = 0.158	ρ 0.209<br>p = 0.136	ρ 0.233<br>p = 0.097	ρ -0.040<br>p = 0.778	-

Table legend. Spearman's rank correlation coefficient (ρ) was used to assess pairwise associations among key angiogenetic cytokines. Each cell reports Spearman's ρ and corresponding *p*-value. Only the lower triangle of the matrix is shown. Significant positive and negative correlations are highlighted in the *Results* section. Correlations related to PlGF are highlighted in bold. * p value < 0.05, ** p value < 0.01.

Abbreviations. PlGF = placental growth factor; EGF = epidermal growth factor; FGF = fibroblasts growth factor; PECAM-1 = platelet endothelial cell adhesion molecule; VEGF = vascular endothelial growth factor; TNF-α = tumor necrosis factor α.

Supplementary Table S2. Univariate Cox regression analysis for overall survival (OS) in the overall cohort (n = 54).

Variable	HR	95% CI	p-value
Age	1.015	0.981-1.050	0.382
KPS < 70	1.148	0.404-3.259	0.796
RPA 3-4	1.868	0.888-3.920	0.100
EGFR amplification	0.471	0.078-2.862	0.414
TERT mutation	1.866	0.189-18.409	0.593
MGMT methylation	0.517	0.254-1.051	0.069
Ki67%	1.019	1.001-1.037	**0.038**
p53%	1.005	0.991-1.018	0.513
Multifocality	6.552	2.723-15.766	**<0.001**
FLAIR volume	0.997	0.992-1.003	0.321
CE volume	0.999	0.982-1.017	0.904
FLAIR only volume	0.996	0.990-1.003	0.259
CE volume only	0.999	0.974-1.024	0.919
FLAIR/CE ratio	0.983	0.867-1.114	0.783
GTR	0.963	0.508-1.826	0.908
Stupp enrolment	0.469	0.216-1.023	**0.057**
PlGF < 13.3 pg/mL	0.741	0.386-1.420	0.366

Table legend. Each covariate was modeled separately. Hazard ratios (HR) > 1 indicate higher hazard (worse survival); HR < 1 indicate lower hazard. Continuous covariates were modeled on their native scale: age per year; Ki-67 and p53 per 1%; volumetric variables per cm^3; FLAIR/CE ratio per unit. Binary covariates were coded as follows: KPS <70 vs ≥70; RPA 3–4 vs RPA 2; EGFR amplification (yes vs no); TERT mutation (yes vs no); MGMT methylation (methylated vs unmethylated); multifocality (multifocal vs unifocal); GTR (gross total resection vs subtotal/biopsy); Stupp enrolment (yes vs no); PlGF <13.3 pg/mL vs ≥13.3 pg/mL (cut-off from maximally selected rank statistic). "FLAIR-only volume" = FLAIR minus CE; "CE-only volume" = CE minus necrosis. Two-sided p-values and 95% confidence intervals are reported.

Supplementary Table S3A. Multivariable survival analysis, extended model

Variable	Overall cohort (n = 54)			RPA 3-4 (n = 33)		
	HR	95% CI	p value	HR	95% CI	p value
Age (years)	1.044	1.005-1.084	**0.026***	1.029	0.967-1.095	0.366
KPS < 70 vs ≥ 70	2.994	0.830-10.802	0.094	2.094	0.176-24.927	0.559
Ki67 %	1.014	0.991-1.037	0.225	1.029	0.978-1.083	0.269
Multifocality	14.5	4.5-46.5	**<0.001****	8.433	2.363-30.088	**0.001****
Stupp enrollment	0.332	0.139-0.798	**0.014***	0.466	0.107-2.028	0.309
PlGF < 13.3 pg/mL	0.402	0.156-1.037	**0.059**	0.199	0.039-1.003	**0.05***

Supplementary Table S3B. Multivariable survival analysis, extended and alternative model.

Variable	Overall cohort (n = 54)			RPA 3-4 (n = 33)		
	HR	95% CI	p value	HR	95% CI	p value
Age (years)	1.034	0.995-1.075	**0.089**	1.024	0.961-1.092	0.463
KPS < 70 vs ≥ 70	2.079	0.488-8.854	0.322	2.044	0.170-24.600	0.573
Ki67 %	1.025	0.998-1.052	**0.065**	1.037	0.982-1.094	0.189
MGMT methylation	0.370	0.147-0.935	**0.035***	0.429	0.070-2.650	0.573
Multifocality	10.922	3.244-36.772	**<0.001****	8.076	2.233-29.206	**0.001****
Stupp enrollment	0.226	0.080-0.635	**0.005****	0.348	0.070-1.733	0.198
PlGF < 13.3 pg/mL	0.381	0.126-1.149	**0.087**	0.213	0.041-1.098	**0.065**

Table legend. Hazard ratios (HR) > 1 indicate higher hazard (worse survival); HR < 1 indicate lower hazard. Continuous covariates were modeled on their native scale: age per year; Ki-67 per 1%. Binary covariates were coded as follows: KPS <70 vs ≥70; MGMT methylation (methylated vs unmethylated); multifocality (multifocal vs unifocal); Stupp enrollment (yes vs no); PlGF <13.3 pg/mL vs ≥13.3 pg/mL (cut-off from the maximally selected rank statistic). Two-sided p-values and 95% confidence intervals are reported.
Significance codes: * p<0.05; ** p≤0.01.

Supplementary Table S4. Alternative multivariable Cox regression models with steroid therapy as a covariate.

Variable	Overall cohort (n = 54)			RPA 3-4 (n = 33)		
	**HR**	**95% CI**	**p value**	**HR**	**95% CI**	**p value**
Age (years)	1.048	1.007-1.090	**0.022***	1.058	0.991-1.129	0.093
KPS < 70 vs ≥ 70	2.185	0.683-6.987	0.188	0.940	0.129-6.849	0.951
Multifocality	11.810	4.087-34.127	**<0.001****	9.441	2.478-35.974	**0.001****
PlGF < 13.3 pg/mL	0.383	0.182-0.803	**0.011***	0.344	0.116-1.018	**0.054**
Steroid therapy at diagnosis	0.977	0.489-1.951	0.948	1.599	0.607-4.210	0.342

Table legend. Hazard ratios (HR) > 1 indicate higher hazard (worse survival); HR < 1 indicate lower hazard. Continuous covariates were modeled on their native scale: age per year. Binary covariates were coded as follows: KPS <70 vs ≥70; multifocality (multifocal vs unifocal); steroid therapy at diagnosis (yes vs no); PlGF <13.3 pg/mL vs ≥13.3 pg/mL (cut-off from the maximally selected rank statistic). Two-sided p-values and 95% confidence intervals are reported.
Significance codes: * p<0.05; ** p≤0.01.

Supplementary Table S5. Adjuvant therapies, toxicity and therapeutic compliance.

Stupp planned [48]	HFRT and TMZ		2 (4.2)
	RT and TMZ		3 (6.3)
	HFRT/TMZ and TMZ		10 (20.8)
	RT/TMZ and TMZ		31 (64.6)
	HFRT only		2 (4.2)
Adjuvant CT (sequential) [48]	Cycles	*Mean ± SD*	4.98 ± 3.59
		Median [Q1, Q3]	5 [2, 6]
		Started	43
		Completed	23 (53.5)
		Interrupted	19 (39.6)
		Not planned due to progression	6 (12.5)
		Toxicity during sequential CT	7 (14.6)
		Progression during sequential CT	12 (25.0)
		Toxicity before sequential CT	1 (2.1)
		Progression before sequential CT	5 (10.4)
		Toxicity without interruption	5 (10.4)
Toxicity [48]	13 (27.1)		
		Hepatopathy	4 (30.8)
		Thrombocytopenia	3 (23.1)
		Infections	2 (15.4)
		DVT	2 (15.4)
		Brain edema	2 (15.4)
Additional therapies	Additional surgeries [54]	23 (42.6)	
	Additional CT [42]	26 (61.9)	
		Regorafenib	13
		TMZ	10
		Bevacizumab	6
		Lomustine	5
	Additional RT [42]	7 (16.7)	
		Gamma Knife	7

Table legend. Number of observations for each variable are indicated in square brackets in the first column. For categorical variable, absolute number and percentages (in brackets) are indicated. AT = adjuvant therapies; CT = chemotherapy; DVT = deep vein thrombosis; HFRT = hypofractionated radiotherapy; PT = platelets; RT = standard radiotherapy; TMZ = temozolomide.

Supplementary Table S6. Survival estimates.

Overall survival (months)	*Median (95% CI)*	17.7 (12.1-23.2)
Progression-free survival (months)	*Median (95% CI)*	8.8 (6.9-10.8)

	Months	Survival (%)	95% CI
OS	6	96.3	91.4 – 100
	12	77.1	66.6 – 89.4
	18	49.6	35.4-62.4
	24	30.3	19.5 – 47.0
PFS	6	76.5	53.2-85.8
	12	23.5	13.1-35.6
	18	11.8	4.8-22.3
	24	4.7	1.5-14.6

Table Legend. Medians (months) with 95% confidence intervals, and Kaplan–Meier survival probabilities (%) with 95% confidence intervals at 6, 12, 18, and 24 months. Abbreviations: OS, overall survival; PFS, progression-free survival.
